# Delaware

**Published:** 1896-04

**Authors:** 


*Delaware.* Of 850 cattle vaccinated as a protective measure against anthrax and returned to infected pastures, no deaths occurred. In this State all cows reacting to the extent of eight-tenths of a degree under the tuberculin-test are isolated.

